# Otitis Media Impacts Hundreds of Mouse Middle and Inner Ear Genes

**DOI:** 10.1371/journal.pone.0075213

**Published:** 2013-10-04

**Authors:** Carol J. MacArthur, Fran Hausman, J. Beth Kempton, Dongseok Choi, Dennis R. Trune

**Affiliations:** 1 Department of Otolaryngology Head and Neck Surgery, Oregon Health & Science University, Portland, Oregon, United States of America; 2 Oregon Hearing Research Center, Oregon Health & Science University, Portland, Oregon, United States of America; 3 Department of Public Health & Preventive Medicine, Oregon Health & Science University, Portland, Oregon, United States of America; University of South Florida, United States of America

## Abstract

**Objective:**

Otitis media is known to alter expression of cytokine and other genes in the mouse middle ear and inner ear. However, whole mouse genome studies of gene expression in otitis media have not previously been undertaken. Ninety-nine percent of mouse genes are shared in the human, so these studies are relevant to the human condition.

**Methods:**

To assess inflammation-driven processes in the mouse ear, gene chip analyses were conducted on mice treated with trans-tympanic heat-killed *Hemophilus influenza* using untreated mice as controls. Middle and inner ear tissues were separately harvested at 6 hours, RNA extracted, and samples for each treatment processed on the Affymetrix 430 2.0 Gene Chip for expression of its 34,000 genes.

**Results:**

Statistical analysis of gene expression compared to control mice showed significant alteration of gene expression in 2,355 genes, 11% of the genes tested and 8% of the mouse genome. Significant middle and inner ear *up*regulation (fold change >1.5, p<0.05) was seen in 1,081 and 599 genes respectively. Significant middle and inner ear *down*regulation (fold change <0.67, p<0.05) was seen in 978 and 287 genes respectively. While otitis media is widely believed to be an exclusively middle ear process with little impact on the inner ear, the inner ear changes noted in this study were numerous and discrete from the middle ear responses. This suggests that the inner ear does indeed respond to otitis media and that its response is a distinctive process. Numerous new genes, previously not studied, are found to be affected by inflammation in the ear.

**Conclusion:**

Whole genome analysis via gene chip allows simultaneous examination of expression of hundreds of gene families influenced by inflammation in the middle ear. Discovery of new gene families affected by inflammation may lead to new approaches to the study and treatment of otitis media.

## Introduction

Otitis media (OM) is a common childhood disorder that results from bacterial or viral infection of the middle ear. Nearly all children experience at least one episode of acute otitis media (AOM), while some children experience recurrent or chronic infections [1]. Episodes of AOM are usually transient and the middle ear inflammation clears within a few days. Inflammatory cytokines are expressed during this phase [2], [3] and in chronic OM (COM), prolonged inflammation places the middle ear and inner ear at risk for tissue destruction and remodeling. Permanent inner ear pathology can result [4], [5], [6] leading to sensorineural hearing loss, delayed speech development, and social and scholastic difficulties. The ability to control these inner ear sequelae is critical to protect children’s hearing, but the multiple processes elicited in the inner ear are only partially understood. It is known that inflammatory factors move through the round window [7] and that cultured spiral ligament fibrocytes produce inflammatory cytokines upon exposure to middle ear pathogens [8], with both mechanisms likely leading to destruction of cochlear tissues. Gene expression array screening studies of mouse AOM and COM mouse showed cochlear tissues express cytokine genes in response to bacterial components or cytokines, or both [9], [10]. This direct induction of a local inflammatory response may underlie the sensorineural hearing loss observed in acute and chronic middle ear inflammation.

Otitis media is known to alter expression of selected cytokine [9], [11], mucin [12], tissue remodeling [11], and ion homeostasis genes [13] in the mouse middle ear (ME) and inner ear (IE). However, whole mouse genome studies of the gene expression in a setting of otitis media have not previously been undertaken. In the acute otitis media mouse model, downregulation of ion homeostasis genes that are involved in ion and water transport and maintenance of tight junctions in the middle ear, in particular aquaporin and Na,K-ATPase genes is seen [13]. Chronic inflammatory middle ear disease can impact inner ear ion and water transport functions and induce tissue remodeling [14]. Recognizing these inner ear mechanisms at risk may identify potential therapeutic targets to maintain hearing during prolonged otitis media. Ninety-nine percent of genes in the mouse are shared in the human [15] so these studies are potentially relevant to the human condition. Using the mouse model for the study of human disease has its limitations. While genes are very similar in the mouse and the human (78% amino acid sequence identity), gene function may diverge between orthologs, making function to phenotype prediction between organisms difficult [16].

Affymetrix^TM^ technology allows the user to study an entire genome in the mouse via a gene chip. Thus, while previous technologies allowed only analysis of selected genes, the chip analysis opens up previously undiscovered genes and pathways for study. The challenge is then to manage the vast amounts of data derived from this technique. The Thompson Reuters Genego MetaCore®program allows analysis of the genetic data obtained from the Affymetrix approach. The data can then be managed with Enrichment Analysis and pathway maps generated for appreciation of new gene pathways at work in a disease process.

The aim of the work described below was to study the entire mouse genome expressed in the inner and middle ear after exposure to inflammation by heat-killed bacteria. The study did accomplish the desired aim.

## Materials and Methods

### Induction of Inflammation

To assess inflammation-driven processes in the mouse ME and IE, gene chip analyses were conducted on mice treated with trans-tympanic heat-killed *Hemophilus influenza (H. flu).* Balb/c mice were screened for absence of ear disease. Animals were sedated with a mixture of ketamine (100 mg/ml; 0.067 mg/gm) and xylazine (20 mg/ml; 0.013 mg/gm). Five microliters of heat-killed *H. flu at* 10^9 ^cfu/ml concentration were trans-tympanically injected into each ear. Untreated Balb/c mice were used as controls. As previously described [13], ME and IE tissues were separately harvested at 6 hours. This study was carried out in strict accordance with the recommendations in the Guide for the Care and Use of Laboratory Animals of the National Institutes of Health. The protocol was approved by the Oregon Health and Science University, Institutional Animal Care and Use Committee, protocol #IS00002622. All surgery was performed under ketamine and xylazine sedation and all efforts were made to minimize suffering.

### RNA Extraction

Ear tissues were dissected in RNAlater (Ambion, Inc., Austin, TX) and stored in RNAlater at −20 degrees until RNA was extracted using Qiagen (Valencia, CA) RNeasy Mini Kit. RNA was extracted using 600 µl of extraction buffer and homogenized using PowerGen 125 homogenizer (Fisher Scientific). RNA was quantified using a NanoDrop 1000 (Thermo Scientific). Due to the need to get adequate RNA for inner ear analysis, each inner ear sample was a pool of 4 animals concentrated using a SpeedVac. For the H. flu exposed inner ear analysis, there were 8 inner ear samples, each a pool of 4 animals, for a total of 32 mice tested (64 ears). For the inner ear control analysis, there were 6 inner ear samples, each a pool of 4 animals, for a total of 24 mice tested (48 ears). For the middle ear analysis, the two ears from each mouse were pooled; there were 9 H flu exposed mice (18 middle ears) and 8 controls (16 ears) run.

Samples were then ethanol precipitated by adding 0.1 volumes of 3 M sodium acetate pH 5.2 and 2.5 volumes of cold 100% ethanol, mixing well and incubating overnight at −20 degrees. Samples were spun at 14,000 rpm at 4°C for 30 minutes, the supernatants removed, and pellets washed twice with 500 µl of cold 80% ethanol. After removing the supernatant, samples were spun again and any excess ethanol removed. Pellets were dried for 1 to 3 minutes in a 42 degree heat-block, then resuspended in 15 µl of RNase-free water and incubated on ice for 30 minutes with occasional vortexing. RNA concentrations ranged from 50–150 ng/ µl. For the middle ear samples, single middle ear RNA isolations were prepared using the same procedure. RNA concentrations for the middle ear ranged from 90–250 ng/ µl.

### Gene Chip Analysis

Middle ear and inner ear samples were processed by the OHSU Core facility on the Affymetrix 430 2.0 Gene Chip for expression of its 34,000 genes. This represents approximately 70% of the entire mouse genome. Gene expression intensity data were transformed into logarithm base 2 and then normalized by quantile normalization [17]. Differential expressions were assessed by linear models. P-values were corrected by false discovery rate (FDR) [18]. All computations were performed using rma [19] and limma [20] packages in R (http://www.R-project.org/). Outliers can affect both numerator (FC) and denominator (standard error) estimates of a test statistics. Since robust multi-array average (RMA) with quantile normalization and moderated t-test (based on an empirical Bayes) were employed, our statistical methodologies, which are some of the most common approaches when analyzing microarray data, are robust methods against outliers.

Data was deposited in the Gene Expression Omnibus repository with accession #GSE49129 (http://www.ncbi.nlm.nih.gov/geo/query/acc.cgi?acc=GSE49129).

### Whole Genome Study

The Affymetrix data was analyzed by the MetaCore^TM^ program from Thomson Reuters. The data set was limited to genes showing >1.5× fold upregulation, or <1.5× downregulation, and FDR [18] adjusted p value <0.05 based on a moderated t-test by an empirical Bayes method [21]. All computations were done in R Statistical language (http://www.R-project.org/). Experimental files were uploaded using a MetaCore™ Parser and stored in the MetaCore™ Data Manager module. The initial list of tags was filtered by applying user-specified threshold(s) to their signal values; the signal values of the tags that have passed the filter were then visualized as linear gauge (“thermometer”) histograms on pathway maps and as colored circles with gradient intensity on the networks.

Enrichment analysis (EA) was performed using the MetaCore™ procedure that consists of mapping gene IDs of the dataset(s) of interest onto gene IDs in entities (terms) of built-in functional ontologies such as pathway maps, networks, diseases, etc. The terms in a given ontology are ranked based on “relevance” in the dataset. The statistical relevance procedure, a p-value of hypergeometric distribution, is calculated as the probability of a match to occur by chance, given the size of the ontology, the dataset and the particular entity. The lower the p-value, the higher is the “non-randomness” of finding the intersection between the dataset and the particular ontology term. That, in turn, translates into a higher ranking for the entity matched. Everything equal, the more genes/proteins belong to a process/pathway, the lower the p-value. The resulting ranking of ontology entities is displayed as a horizontal bar histogram in which each bar represents a particular entity and has a link to the relevant pre-built map or network, or to the network building procedure. In EA, multiple proprietary ontologies (canonical pathway maps, cellular processes, toxicities, disease biomarkers etc.), and public ontologies such as Gene Ontology (http://geneontology.org/) (cellular processes, protein functions, localizations) are used. The program also performs interactome analysis with the interactome tool that allows a user to estimate interconnectedness of an experimental dataset (density of interactions), find statistically significant interactions in the set and perform enrichment of the dataset with protein classes.

Pathway maps were then selected from the top 25 pathways list in our dataset in the middle ear and inner ear pathways. The goal of pathway based analyses is to determine the interconnectivity within a given set of data and how this is biologically relevant. Pathway analysis is also essential in determining the relevance of genes/proteins that alone may not be significantly altered but if they exist in a signaling pathway with a set of other altered genes/proteins then collectively, they have a profound biological outcome.

### Ion Homeostasis Gene Evaluation

Because numerous ion channels occur in both the middle ear and inner ear [22] we also evaluated the impact of middle ear inflammation on these transporters and channels. There is no cellular process group for ion homeostasis within the Gene Ontology analysis software, but their function is critical to both the middle ear for fluid control and the inner ear for endolymph regulation. Therefore, the entire list of significantly affected genes was scanned for genes that have relevance to tight junctions, gap junctions, and channels involved in the passage of sodium, potassium, calcium, zinc, and other ions.

### Correlation of Affymetrix and qRT-PCR Results

The qRT-PCR technique is often used to assess cytokine gene expression in the middle and inner ear during otitis media. However, studies have not been done that compare the PCR gene expression results with those from the Affymetrix Gene Chip technology. This is important in order to correlate results using the two methods. Therefore, qRT-PCR was performed on middle and inner ear tissues from 16 Balb/c mice inoculated with H flu and tissues collected 6 hours later. Untreated mice (N = 16) were used as controls. The qRT-PCR protocol followed previously reported techniques [14].

Induction of inflammation and RNA extraction from IE and ME tissues was performed as described above. Six hours following inoculation, 16 mice were euthanized, the bullae harvested, and the inner and middle ear tissues dissected, using 8 mice for the IE and 8 for the ME. As previously described [13], ME and IE tissues were separately harvested at 6 hours. Left and right middle ear tissues were combined for each mouse and processed together to provided 8 independent middle ear samples. Middle ears from 8 control mice were processed in parallel. The remaining 8 inoculated mice (and 8 controls) were euthanized and inner ears isolated. Left and right inner ears for each mouse were combined and processed together, thus providing 8 independent inner ear samples. These inner ear issues were processed similar to the middle ear components above, along with 8 control inner ear samples.

### Quantitative RT-PCR Analyses

The middle ear and inner ear mRNA was processed for quantitative RT-PCR of our standard profile of 8 inflammatory cytokine genes previously reported to be major components of the middle ear inflammatory response [13], [14]. The method utilized custom PCR Arrays (RT2 Profiler PCR Array System, SABiosciences Corp, Frederick, MD) already optimized for reaction conditions, primers, and probes. These included interleukins (IL-1α, IL-1β, IL-6, IL-10), macrophage inflammatory proteins (MIP-1α or Ccl3, MIP-2α or Cxcl2), tumor necrosis factor-α (TNFα), and keratinocyte-derived chemokine (KC or Cxcl1). Real-time RT-PCR studies used an ABI Step One Plus system (Carlsbad, CA) as previously reported [13], [14].

Fold change of these 8 cytokines were compared for the Affymetrix and PCR methods. Statistical correlations of cytokine expression between the two methods were determined by regression analysis for middle ear and inner ear results.

## Results

To obtain an understanding of how many genes from the mouse genome were present in the normal mouse middle and inner ear, we used the criteria that to count a gene as being present, at least half of the transcripts had to be positive on the array. Furthermore, half of the positive transcripts had to occur in 50% of animal samples run. Of the 21,815 unique identified genes on the gene chip, 13,495 were identified in the middle ear (62%) and 17, 485 in the inner ear (80%).

Results were statistically analyzed for expression of each gene against control (untreated) mice. Significant alteration of gene expression was seen in 2,355 genes, 11% of the genes tested and 8% of the mouse genome. A list of these genes with fold changes, log_2_ (FC) and standard errors (error bars) in the log_2_ scale is provided in Table S1 (ME and IE).

**Figure 1:**
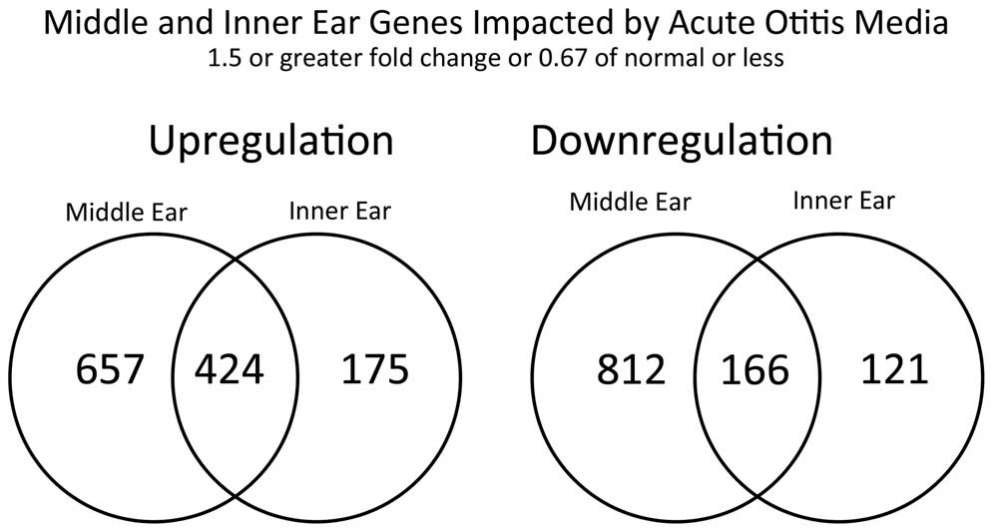
Middle and inner ear genes affected by acute inner ear inflammation. Middle and inner ear genes affected by acute inner ear inflammation, >1.5× upregulation or <1.5× downregulation. Note that while there is overlap between the shared inner and middle ear genes up- or downregulated, there is a high percentage of unique genes in both categories.

Significant ME and IE *up*regulation (fold change >1.5, FDR adjusted p<0.05) was seen in 1,081 and 599 genes respectively (Fig. 1). Significant ME and IE *down*regulation (fold change <1.5 and DR adjusted p<0.05) was seen in 978 and 287 genes respectively (Fig. 1). Twenty-nine percent of IE genes upregulated were unique to the IE, while 60% were unique to the ME. Forty-two percent of the IE genes downregulated were unique to the IE, while 83% were unique to the ME.

### Enrichment by Protein Function

The dataset genes and protein/gene interactions were limited to fold change >1.5× upregulation or <1.5× downregulation, and FDR adjusted p<0.05. After this cutoff, the following protein enrichment interactions were judged significant: ME upregulation 965, IE upregulation 566, ME downregulation 861, and IE downregulation 258. Table 1 lists the number and percentages of middle and inner ear genes that were either upregulated or downregulated as analyzed by interactome analysis. Note that enzymes, ligands and receptors were up- and downregulated the greatest in our ME and IE datasets. While more gene expression was altered over controls in the ME, there are still a large number of IE genes showing alteration of expression.

**Table 1:** Interactome Analysis of ME and IE Genes.

	Middle Ear				Inner Ear			
Protein Class	ME upreg	ME % of upreg	ME downreg	ME % of downreg	IE upreg	IE % of upreg	IE downreg	IE % of downreg
Enzymes	122	12.64%	107	12.43%	59	10.42%	39	12.84%
Kinases	43	4.46%	14	1.63%	13	2.30%	2	3.04%
Ligands	79	8.19%	32	3.72%	57	10.07%	11	5.79%
Phosphatase**s**	13	1.35%	6	0.70%	4	0.71%	1	0.97%
Proteases	38	3.94%	21	2.44%	29	5.12%	10	3.28%
Receptors	115	11.92%	52	6.04%	75	13.25%	22	9.07%
Transcription factors	71	7.36%	43	4.99%	33	5.83%	14	5.84%
Other	487	50.47%	587	68.18%	297	52.47%	159	59.41%
n	965		861		566		258	

Interactome analysis: performed with limiting the dataset to named genes and protein/gene interactions *in our data set*, fold change >1.5× upregulation and <1.5× downregulation and p<0.05.

### Ion Homeostasis Genes

After limiting the dataset to genes with 1.5 fold change, and FDR adjusted p<0.05, ion homeostasis genes showing significant up- or down-regulation were extracted. Table 2 summarizes the impact of middle ear inflammation on various ion homeostasis genes in the middle ear and inner ear. Generally, a gene affected in the middle ear was impacted the same way in the inner ear. This is particularly evident for genes that were expressed at higher levels due to inflammation. Nearly every gene significantly upregulated in the middle ear was also upregulated in the inner ear. Downregulated genes in the inner ear were fewer than in the middle ear, suggesting suppressed expression was less common in the inner ear during middle ear disease.

**Table 2:** Impact of Middle Ear Inflammation on Ion Homeostasis Genes in ME and IE.

Gene Upregulated	ME	IE	Gene Downregulated	ME	IE
aquaporin 3	X	X	aquaporin 5	X	X
ATPase, Na+/K+ transporting, alpha 4 polypeptide	X	X	aquaporin 7	X	
transporter 1, ATP-binding cassette, sub-family B (MDR/TAP)		X	ATPase, (Na+)/K+ transporting, beta 4 polypeptide	X	
chloride channel, calcium activated, regulator 1		X	ATPase, H+ transporting, lysosomal V1 subunit B1	X	
chloride channel, calcium activated, regulator 2		X	ATPase, H+ transporting, lysosomal V1 subunit C2	X	
claudin 1		X	calcium channel, voltage-dependent, alpha2/delta subunit 1		X
claudin 2		X	calcium channel, voltage-dependent, beta 3 subunit	X	
claudin 4	X	X	claudin 5	X	X
claudin 7	X	X	claudin 8	X	
potassium channel tetramerisation domain containing 1	X	X	claudin 19		X
potassium inwardly-rectifying channel, subfamily J, member 15	X	X	claudin 22	X	X
potassium voltage-gated channel, Isk-related subfamily, gene 3	X	X	claudin 23	X	
potassium voltage-gated channel, Isk-related subfamily, gene 4	X		gap junction protein, beta 5	X	
potassium voltage-gated channel, shaker-related subfamily, member 5	X		potassium channel tetramerisation domain containing 4		X
potassium voltage-gated channel, subfamily H (eag-related), member 1	X	X	potassium channel tetramerisation domain containing 12b	X	
solute carrier family 1 (neuronal/epithelial high affinity glutamatetransporter, system Xag), member 1	X	X	potassium channel tetramerisation domain containing 15	X	
solute carrier family 2 (facilitated glucose transporter), member 6	X	X	potassium channel, subfamily K, member 2	X	
solute carrier family 4 (anion exchanger), member 8		X	potassium channel, subfamily K, member 3	X	
solute carrier family 5 (sodium/glucose cotransporter), member 1	X	X	potassium inwardly-rectifying channel, subfamily J, member 16	X	
solute carrier family 6 (neurotransmitter transporter), member 14	X	X	potassium inwardly-rectifying channel, subfamily J, member 8	X	
solute carrier family 7 (cationic amino acid transporter, y+ system),member 1	X		potassium large conductance calcium-activated channel,subfamily M, beta member 1	X	
solute carrier family 7 (cationic amino acid transporter, y+ system),member 2	X	X	potassium large conductance calcium-activated channel,subfamily M, beta member 2	X	
solute carrier family 7 (cationic amino acid transporter, y+ system),member 6	X		potassium large conductance calcium-activated channel,subfamily M, beta member 4	X	
Solute carrier family 7, member 6 opposite strand	X		potassium voltage-gated channel, Isk-related subfamily, gene 2	X	X
solute carrier family 7 (cationic amino acid transporter, y+ system),member 8		X	potassium voltage-gated channel, Isk-related subfamily,member 1	X	
solute carrier family 7 (cationic amino acid transporter, y+ system),member 11	X		sodium channel, voltage-gated, type III, alpha	X	
solute carrier family 9 (sodium/hydrogen exchanger), member 3	X	X	solute carrier family 2 (facilitated glucose transporter),member 12	X	
solute carrier family 10 (sodium/bile acid cotransporter family), member 6	X	X	solute carrier family 4, sodium bicarbonate transporter-like,member 11	X	X
solute carrier family 11 (proton-coupled divalent metal ion transporters), member 1	X		solute carrier family 5 (inositol transporters), member 3	X	
solute carrier family 11 (proton-coupled divalent metal ion transporters), member 2	X		solute carrier family 6 (neurotransmitter transporter, dopamine), member 3		X
solute carrier family 15 (oligopeptide transporter), member 1		X	solute carrier family 6 (neurotransmitter transporter, serotonin), member 4		X
solute carrier family 15, member 3	X	X	solute carrier family 8 (sodium/calcium exchanger), member 3		X
solute carrier family 16 (monocarboxylic acid transporters), member 3	X		solute carrier family 9 (sodium/hydrogen exchanger), member 2	X	
solute carrier family 16 (monocarboxylic acid transporters), member 12		X	solute carrier family 15 (H+/peptide transporter), member 2	X	
solute carrier family 25 (mitochondrial carrier, phosphate carrier),member 25	X		solute carrier family 16 (monocarboxylic acid transporters), member 11	X	
solute carrier family 26, member 3		X	solute carrier family 16 (monocarboxylic acid transporters), member 13	X	
solute carrier family 28 (sodium-coupled nucleoside transporter), member 3	X	X	solute carrier family 22 (organic cation transporter), member 2		X
solute carrier family 30 (zinc transporter), member 2	X	X	solute carrier family 23 (nucleobase transporters), member 1	X	
solute carrier family 34 (sodium phosphate), member 2	X	X	solute carrier family 25, member 35	X	
solute carrier family 39 (metal ion transporter), member 8	X		solute carrier family 44, member 3	X	
solute carrier family 39 (zinc transporter), member 4	X	X	solute carrier family 45, member 4	X	
solute carrier family 39 (zinc transporter), member 14	X	X	solute carrier family 46, member 1	X	X
			solute carrier organic anion transporter family, member 1a4	X	
			solute carrier organic anion transporter family, member 1c1	X	
			solute carrier organic anion transporter family, member 2b1	X	

The two major groups of homeostasis genes affected were the aquaporins and tight junction claudins. These gene products are responsible for sealing tissue compartments (middle ear mucosa, inner ear endolymphatic chambers). A significant number of ion transporting genes also were impacted. These included those for moving potassium, calcium, and sodium, as well as a number of other ions. These results suggest that maintenance of fluid filled (inner ear) or fluid free (middle ear) compartments is compromised during middle ear inflammation.

### Top 25 Pathways in Our Dataset

We examined the top 25 pathways in our dataset to look for novel pathways that fit with our most affected genes (Table 3). Table 3 lists the top 25 pathways for the middle ear tissue (vs. controls), and corresponding rank order in the inner ear tissues (vs. controls). Out of the top 25 pathways, immune response pathways comprise 16, development 3, cytokine production 2, apoptosis 2, bacterial infection 1, transcription 1. Immune response pathways in the top 25 pathways from our dataset include expected pathways of proinflammatory cytokine signaling (IL-1 and IL-17 signaling), innate immune system signaling (TLR signaling), but also previously unreported immune pathways for the ear (High-mobility group (HMG) B1 release, HMGB1/TLR signaling, Role of HMGB1 in dendritic cell maturation and migration, HMGB1/Receptor for Advanced Glycan Endproducts (RAGE) signaling, interferon (IF) antiviral pathway, IF-mediated glucocorticoid regulation, IF in innate immune response, Histamine H1 receptor signaling in immune response, Oncostatin M signaling via JAK-Stat in human cells, CD-40 signaling. Development pathways include Pigment epithelium-derived factor (PEDF) signaling, Granulocyte macrophage colony-stimulating factor receptor (GM-CSF) signaling, Regulation of epithelial-to-mesenchymal transition (EMT) and Thrombopoetin signaling via JAK-STAT pathway. Apoptosis pathways include: a proliferation-inducting ligand (APRIL) and B-cell activating factor (BAFF) signaling and Anti-apoptotic TNFs/NF-kB/Bcl-2 pathway (#1 for ME data). The one transcription pathway in our top 25 is the NF-kB signaling pathway, a transcription factor that is activated by cytokines and bacterial or viral products.

**Table 3:** Pathway Analysis – Top 25 matching with our dataset.

		Middle Ear				Inner Ear			
Pathway maps	Pathway Rank		p-value	# Differentially expressed genes	# Genes in pathway	Pathway Rank	p-value	# Differentially expressed genes	# Genes in pathway
Apoptosis and survival_Anti-apoptotic TNFs/NF-kB/Bcl-2 pathway	1		1.57E-13	20	41	34	6.51E-05	8	41
Bacterial infections in CF airways	2		5.63E-13	23	58	3	4.90E-12	17	58
Development_PEDF signaling	3		9.44E-13	21	49	9	6.56E-09	13	49
Immune response_IL-17 signaling pathways	4		1.17E-11	22	60	1	3.68E-15	20	60
Immune response_Bacterial infections in normal airways	5		1.56E-11	20	50	10	8.59E-09	13	50
Cytokine production by Th17 cells in CF	6		1.04E-10	17	39	2	5.03E-14	16	39
Immune response_HMGB1/RAGE signaling pathway	7		4.74E-10	19	53	4	1.28E-11	16	53
Immune response_CD40 signaling	8		5.33E-10	21	65	12	3.15E-08	14	65
Immune response_IL-1 signaling pathway	9		1.02E-09	17	44	6	1.51E-09	13	44
Transcription_NF-kB signaling pathway	10		1.11E-09	16	39	26	5.26E-06	9	39
Development_GM-CSF signaling	11		1.27E-09	18	50	32	4.45E-05	9	50
Immune response_TLR signaling pathways	12		1.40E-09	19	56	13	3.80E-08	13	56
Immune response_Oncostatin M signaling via JAK-Stat in mouse cells	13		2.19E-09	11	18	14	5.96E-08	8	18
Immune response_Antiviral actions of Interferons	14		2.64E-09	18	52				
Development_Regulation of epithelial-to-mesenchymal transition (EMT)	15		2.68E-09	20	64	29	1.01E-05	11	64
Immune response_Role of HMGB1 in dendritic cell maturationand migration	16		3.80E-09	13	27	18	1.68E-07	9	27
Cytokine production by Th17 cells in CF (Mouse model)	17		7.05E-09	17	49	5	4.54E-11	15	49
Immune response_Oncostatin M signaling via JAK-Stat inhuman cells	18		1.02E-08	11	20	17	1.62E-07	8	20
Immune response_Histamine H1 receptor signaling in immune response	19		3.78E-08	16	48	8	4.97E-09	13	48
Immune response_MIF-mediated glucocorticoid regulation	20		3.79E-08	11	22	11	2.07E-08	9	22
Immune response_MIF in innate immunity response	21		1.35E-07	14	40	15	6.54E-08	11	40
Immune response_HMGB1 release from the cell	22		1.93E-07	14	41	45	4.47E-04	7	41
Immune response_HMGB1/TLR signaling pathway	23		2.50E-07	13	36	20	2.37E-07	10	36
Development_Thrombopoetin signaling via JAK-STAT pathway	24		5.01E-07	10	22	35	7.54E-05	6	22
Apoptosis and survival_APRIL and BAFF signaling		25	7.30E-07	13	39	60	2.01E-03	6	39

**Figure 2:**
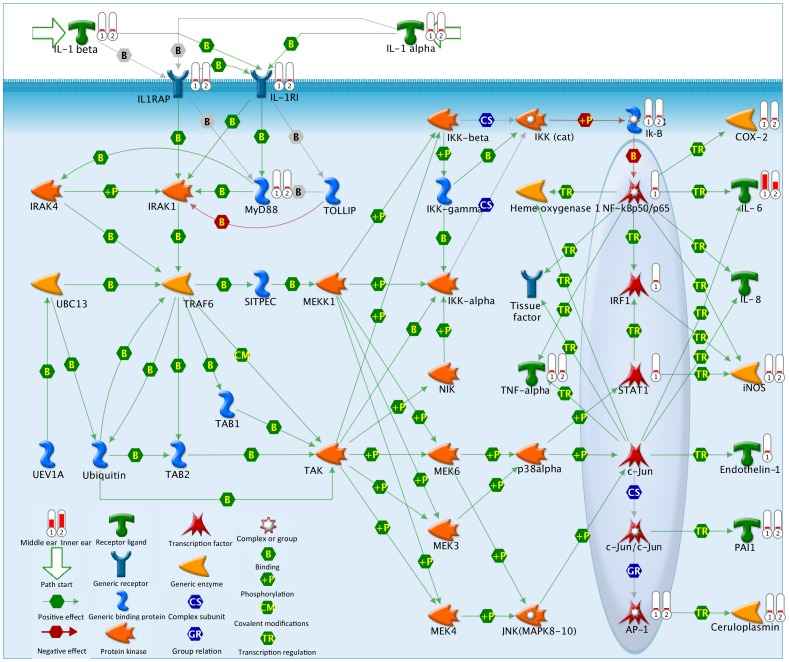
IL-1 Pathway Map. IL-1 Pathway Map, showing all genes in theIL-1 gene pathway and the amount of upregulation seen in our dataset by the histogram bars (Cox-2, IL-6, iNOS, endothelin-1, Pal1, STAT1, IRF1, NF-kB p50/p65, TNFα, Myd88, IL-1Rl, IL1RAP, IL-1α, IL-1β). Highest fold change activity seen in IL-6 for both ME and IE. (Data summary from Genego.)

**Figure 3:**
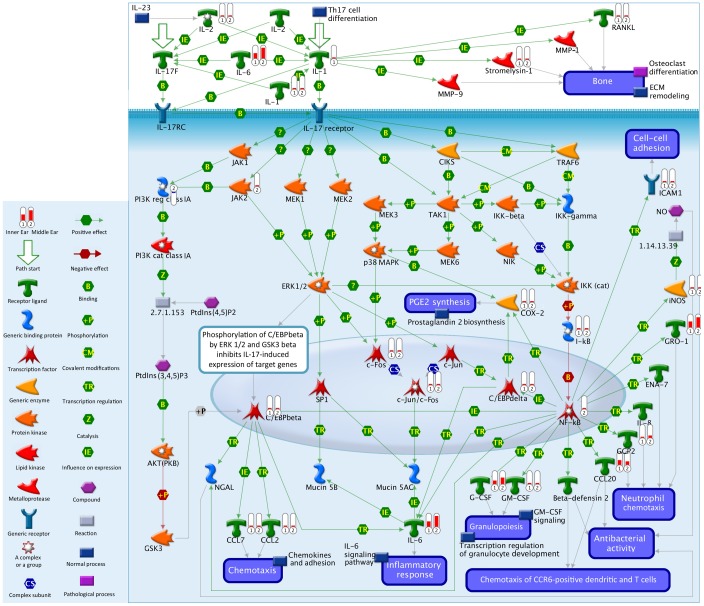
IL-17 pathway map. IL-17 pathway map, showing all genes in the IL-1 gene pathway and the amount of upregulation seen in our dataset by the histogram bars (G-CSF, GM, CSF, c-FOS, CCL2 and 7, iNOS, GRO-1, IL-2, IL-1, IL-8, RANKL, Stromelysin, JAK2, C/EPbeta). Highest fold change activity seen in IL-6 for both ME and IE. (Data summary from Genego.)

The IL-1 pathway was ranked #9 and #6 for the ME and IE data from our experiment, respectively. Figure 2 shows the entire IL-1 pathway along with the fold change data from our ME and IE data, in histograms on the figure, compared to controls. Note that the histogram bar shows high level of activity for IL-6. Table 4 summarizes the actual IL-1 pathway fold change values for our dataset, showing the high levels of IL-6 fold change for both ME and IE data. While all cytokines levels were elevated in this pathway, other highly elevated objects in the pathway were COX-2 (Ptgs2 gene) and AP-1 (Fosl1 gene). Figure 3 shows the pathway map for IL-17, #4 and #1 in the Pathway Maps listing from our dataset (for the ME and IE data, respectively). Table 5 lists the IL-17 fold change values for our dataset, again showing the high levels of IL-6 fold change for both ME and IE data. Large fold change values were seen in the IL-17 pathway in chemokines, interleukins, G-CSF and Cox-2 (PTGS2 gene).

**Table 4:** IL-1 pathway fold change values.

Pathway object	Object type	Full Name	Genes	ME fold change	p-value	IE fold change	p-value
IL-1 beta	Receptor Ligand	interleukin 1 beta	IL1b	15.39	7.12E-20	5.15	1.50E-12
IL-1 alpha	Receptor Ligand	interleukin 1 alpha	IL1a	10.25	6.28E-15	3.30	5.96E-09
IL1RAP	Receptor	interleukin 1 receptor accessory protein	Il1rap	3.02	4.64E-11	2.10	3.82E-03
IL-1R1	Receptor	interleukin 1 receptor, type I	IL1r1	2.08	7.90E-09	2.44	2.22E-03
MyD88	Binding Protein	myeloid differentiation primary responseprotein 88	Myd88	2.81	7.52E-17	1.77	1.69E-06
I-kB	Binding Protein	I-kB proteins	Nfkbia	3.85	6.54E-17	2.14	1.97E-10
			Nfkbib	1.66	2.01E-04	1.74	7.16E-06
			Nfkbie	2.20	4.17E-12		
NFkB p50/p65	Transcription Factor	nuclear factor of kappa light polypeptide gene enhancer in B cells	Nfkb1	1.92	7.15E-11		
			Rela	1.50	7.59E-10		
COX-2	Enzyme	Prostaglandin-endoperoxide synthase 2	Ptgs2	26.05	1.20E-13	6.89	1.93E-09
IL-6	Receptor Ligand	interleukin 6	Il6	306.66	1.13E-20	162.24	1.62E-18
TNF-alpha	Receptor Ligand	Tumor necrosis factor	Tnf	4.90	1.38E-15	2.78	3.61E-09
IRF1	Transcription Factor	Interferon regulatory factor 1	Irf1	1.71	5.42E-09		
STAT1	Transcription Factor	Signal transducer and activator of transcription 1	Stat1	1.85	2.64E-07		
iNOS	Enzyme	nitric oxide synthase 2, inducible	Nos2	4.61	4.51E-11	2.15	1.63E-06
Endothlin-1	Receptor Ligand	Endothelin 1	Edn1	1.61	5.66E-08		
PAI1	Receptor Ligand	Plasminogen activator inhibitor 1	Serpine1	2.40	9.20E-08	1.88	1.07E-05
AP-1	Transcription Factor	AP-1 protein complexes	Atf2	1.50	2.14E-05		
			Fos	5.84	2.60E-13	3.48	9.08E-07
			Fosl1	24.78	3.68E-11	6.71	7.33E-11
			Fosl2	3.33	1.37E-15	1.60	1.56E-07
			Junb	3.40	4.84E-17	1.99	4.57E-09
Ceruloplasmin	Enzyme	Ceruloplasmin	Cp	1.80	1.03E-06	2.73	1.13E-05

**Table 5:** IL-17 pathway fold change values.

Pathway object	Object Type	Full Name	Genes	ME fold change	p-value	IE fold change	p-value
C/EBPbeta	Transcription factor	CCAAT/enhancer-binding protein beta	Cebpb	2.7647534	7.336E-15	2.4452081	7.485E-10
C/EBPdelta	Transcription factor	CCAAT/enhancer-binding protein delta	Cebpd	3.9557347	9.884E-17	2.4882632	1.758E-12
CCL2	Receptor ligand	C-C motif chemokine 12	Ccl12	3.9987144	3.336E-09	4.344023	1.319E-11
CCL20	Receptor ligand	C-C motif chemokine 20	Ccl20	93.818	2.308E-20	138.14658	2.96E-17
CCL7	Receptor ligand	C-C motif chemokine 7	Ccl7	24.522788	3.668E-14	11.711994	2.808E-12
COX-2 (PTGS2	Generic enzyme	Prostaglandin G/H synthase 2	Ptgs2	26.047008	1.197E-13	6.8869924	1.928E-09
G-CSF	Receptor ligand	Granulocyte colony-stimulating factor	Csf3	185.0193	1.945E-20	97.682787	4.163E-17
GCP2	Receptor ligand	C-X-C motif chemokine 5	Cxcl5	64.833257	5.286E-23	16.218563	1.159E-08
GM-CSF	Receptor ligand	Granulocyte-macrophagecolony-stimulating factor	Csf2	14.880254	1.57E-15	13.199347	6.579E-12
GRO-1	Receptor ligand	C-X-C motif chemokine 3	Cxcl3	302.08509	2.262E-20	170.62942	2.125E-18
I-kB	Generic binding protein		Nfkbia	3.8538761	6.539E-17	2.1419256	1.974E-10
I-kB	Generic binding protein		Nfkbib	1.6639867	0.0002008	1.7365158	7.161E-06
ICAM1	Generic receptor	Intercellular adhesion molecule 1	Icam1	5.104816	1.168E-19	3.6836136	2.439E-11
IL-1beta	Receptor ligand	Interleukin-1 beta	Il1b	15.387494	7.118E-20	5.147765	1.502E-12
IL-17	Receptor ligand	Interleukin-17A	Il17a			1.6200989	0.0008791
IL-23	Receptor ligand	Interleukin-23	Il12B	3.3397873	2.999E-09	2.150198	8.216E-07
IL-23	Receptor ligand	Interleukin-23A	Il23A	4.1948946	1.186E-08	1.7845918	8E-07
IL-6	Receptor ligand	Interleukin-6	Il6	306.66243	1.134E-20	162.2436	1.618E-18
RANKL(TNFSF11)	Receptor ligand	Tumor necrosis factor ligandsuperfamily member 11	Tnfsf11	4.5062763	1.304E-12	3.495858	2.203E-05
Stromelysin-1	Metalloprotease	Stromelysin-1	Mmp3	10.689979	4.324E-13	14.69291	4.203E-11
c-FOS	Transcription factor	Proto-oncogene c-Fos	Fos	5.8445803	2.596E-13	3.478137	9.082E-07
INOS	Generic enzyme	Nitric oxide synthase, inducible	Nos2	4.6079374	4.511E-11	2.1456909	1.635E-06

**Figure 4:**
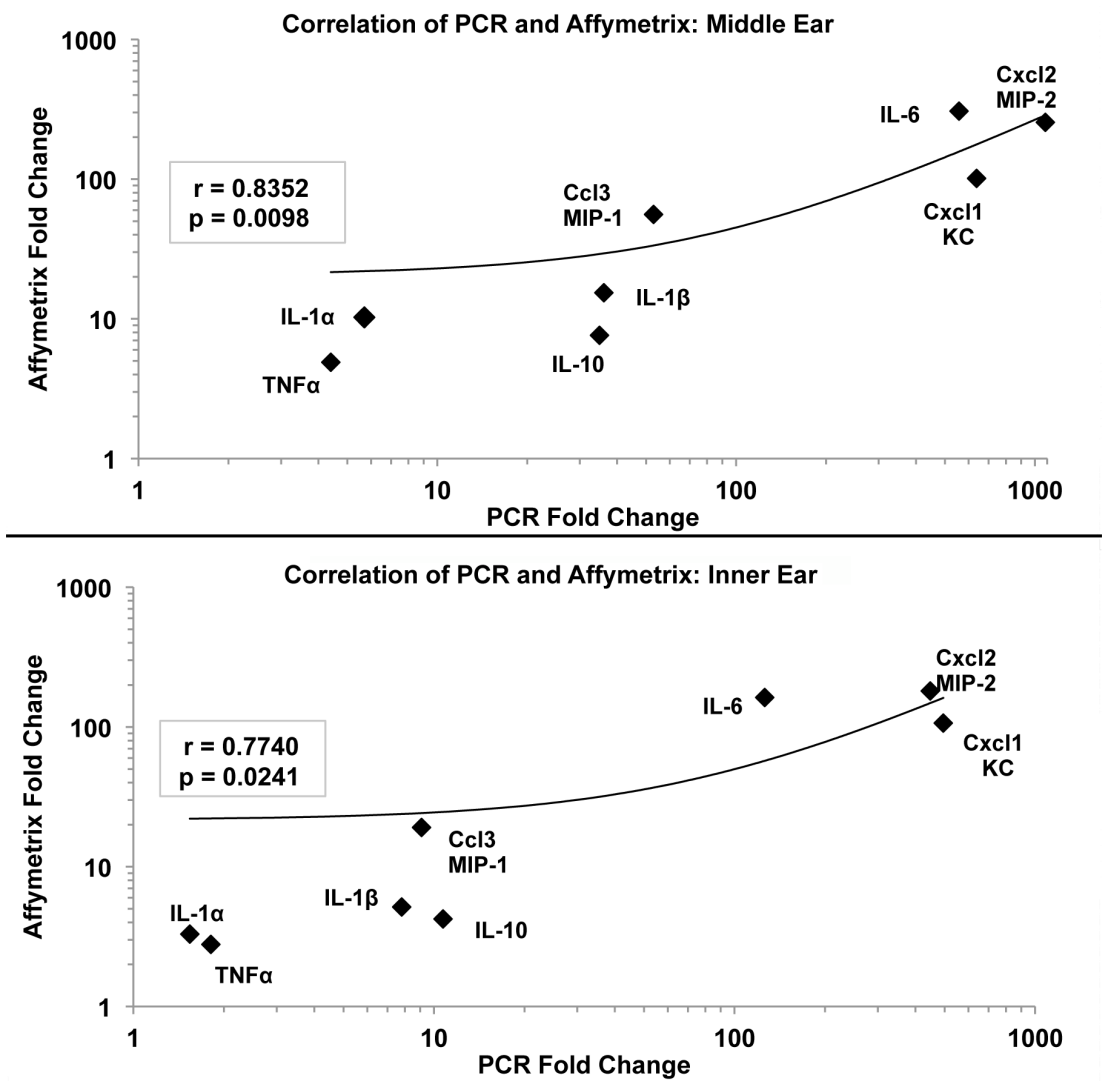
Cross comparison of cytokine gene expression measured by Affymetrix Gene Chip and qRT-PCR. Middle ear fold change analyses (top) showed a significant correlation between the two methods (p = 0.0098). Inner ear analyses (bottom) showed a similar pattern of cytokine expression that also was significantly correlated between the two methods (p = 0.0241).

A comparison was done of qRT-PCR and Affymetrix techniques for eight of the commonly tested cytokines in the middle and inner ear. Cross comparison of the Affymetrix and qRT-PCR results showed similar fold changes of cytokine gene expression (Fig. 4). Middle ear tissues processed with the two methods showed remarkable similarity in fold changes for the respective cytokines with a correlation of 0.8352 (p = 0.0098). TNFα and IL-1α showed the least expression due to the inflammation (< 10 fold), MIP-1, IL-1β, and IL-10 were grouped together in a moderate response (10–100 fold), while KC, MIP-2, and IL-6 showed the greatest reactive expression (500–1,000 fold). Very similar results were seen for the inner ear cytokine gene expression (Fig. 4). Not only were the same cytokines expressed in similar amounts as the middle ear, their groupings were virtually identical. The correlation of inner ear cytokine expression between the two methods also was statistically significant, showing a correlation of 0.7740 (p = 0.0241). These results reveal a strong cross correlation between the two methods of assessing gene expression in the middle and inner ear, showing not only significantly comparable findings, but also similar patterns in the cytokine genes involved.

## Discussion

These studies show hundreds of ME and IE genes are affected by inflammation. While otitis media is widely believed to be an exclusively middle ear process with little impact on the inner ear, the IE gene changes noted in this study were numerous and discrete from the ME responses. This suggests that the IE does indeed respond to otitis media and that the IE response to ME inflammation is a distinctive process from that occurring in the ME. Numerous new genes, previously not studied, were found to be affected by inflammation in the ear. Of the top 25 pathway maps from our data, 15 were immune response, 4 development/signaling, 2 apoptosis/survival, 2 cytokine production, 1 transcription and 1 bacterial infection.

Numerous ion homeostasis genes were up- or down-regulated in the IE and ME as a result of the middle ear inflammation. Generally genes were impacted similarly in the inner and middle ear. This is particularly evident for genes that are expressed at higher levels due to inflammation. Nearly every gene significantly upregulated in the middle ear was also upregulated in the inner ear. The fact that downregulated genes were fewer in the inner ear suggests that suppressed expression is more likely in the middle ear due to local inflammatory disease processes. Recent research has shown that middle ear ion transport is extensive [22] and is probably involved in clearing of middle ear fluid accumulation during otitis media [13], [23], [24], [25]. This direct effect of inflammation on middle ear ion homeostasis functions also is seen in the inner ear [14], [26], [27], [28]. This disruption of ion transport by inflammation has been observed in other systems, as well [29].

New genes of interest also were encountered by our pathway analysis. For example, Interleukin 17 (IL-17) is a cytokine that acts as a potent mediator in delayed-type reactions by increasing chemokine production in various tissues to recruit monocytes and neutrophils to the site of inflammation. The IL-17 family functions as a proinflammatory cytokine that responds to the invasion of the immune system by extracellular pathogens and induces destruction of the pathogen’s cellular matrix. While increased IL-17 expression was shown in the rabbit ME after exposure to gastric contents, increased IL-17 has not been reported secondary to otitis [30]. The anti-apoptotic TNF/NF-kB/Bcl-2 pathway may be at play in the ME inflammatory cascade as evidenced by its position as pathway rank #1 for the ME. Also interesting is the commonality of inflammatory pathways and tumor-signaling pathways seen in our pathway map list (Oncostatin M signaling, Epithelial-mesenchymal transition (EMT) regulation, HMGB1/RAGE, APRIL & BAFF signaling, etc.). The High-mobility group (HMG) proteins have activity as chemokines and antibodies to HMG proteins can be found in autoimmune disease patients**.** Oncostatin M, a pleiotropic cytokine is active in both pro- and anti-inflammatory pathways and IL-6 expression, interestingly. While its role has been described in airway inflammation [31], it has not been described in otitis. The GM-CSF signaling pathway was noted to be active in the pathway map (#11, 32 respectively for ME and IE). GM-CSF is part of the immune/inflammatory cascade, with action on the inflammatory response, cell differentiation, cell proliferation and anti-apoptosis pathways. GM-CSF has recently been ascribed a role in immunity against pathogens via its action in stimulating production of granulocytes and monocytes/macrophages, as well as dendritic cell subsets [32]. GM-CSF enhances innate host defenses against microbial pathogens, and increases absolute numbers of circulating innate immune effector cells by accelerating bone marrow production and maturation [33]. As GM-CSF acts on bone marrow cells via hematopoiesis, its activity in our inflammation model implies that GM-CSF, and possibly other genes, may be at work locally in the bulla bone/bone marrow of the ear. The possible role of these pathways in the inflammation seen with otitis media bears further study.

The cross correlation showed PCR and Affymetrix gene chip measures were remarkably similar and significantly correlated. This should offer some confidence in the evaluation of research reports using only one of the two methods. It was also remarkable that the middle ear and inner ear tissues showed virtually identical patterns of increased cytokine expression during the middle ear inflammation. This finding is also noteworthy in that the different ear region tissues respond similarly to the inflammatory factors, emphasizing the common innate immune response mechanisms shared by distinctly different cell types. Limitations of this approach are that the classification tools used in GeneGo are biased towards previously characterized pathways. So, this approach to analysis of the data set does not allow for uncovering novel pathways and mechanisms, but it does allow identification of novel genes involved in a disease process that were not previously recognized. Also, the assay is performed at the level of gene expression, rather than protein or functional assays. Further analysis of impact on actual protein levels seen in relation to gene expression changes could confirm that these pathways are impacting inner and middle ear functions. In addition, the study represents a snap shot of the disease process. The use of the early time point undoubtedly highlights the innate immune response to the acute inflammatory state of early otitis media. While the study shows inner ear activity in response to inflammation in the middle ear, the majority of infants would experience several episodes of AOM which usually resolve without significant inner ear sequelae or sensorineural hearing impairment.

## Conclusion

Whole genome analysis via gene chip allows simultaneous examination of expression of hundreds of gene families influenced by inflammation in the ME. Discovery of new gene families affected by inflammation may lead to new approaches to the study and treatment of otitis media.

## Supporting Information

Table S1 An entire list of the significant genes with fold changes, log_2_ (FC) and standard errors (error bars) in the log_2_ scale is provided.
(XLSX)
